# Swiss cheese interventricular septum presenting with catastrophic stroke: the potential role of ECG-gated CTA

**DOI:** 10.1259/bjrcr.20210069

**Published:** 2021-11-12

**Authors:** Weibo Fu, Lauren Gates, Mohamed Issa, William B. Bates, J. Jeff Carr, Wael Aljaroudi, Vincent Sorrell, Michael A. Winkler

**Affiliations:** 1 Medical College of Georgia at Augusta University, Augusta, Georgia; 2 Vanderbilt University Medical Center, Nashville, Tennessee; 3 University of Kentucky College of Medicine, Lexington, Kentucky

## Abstract

Ventricular septal defect is a common congenital cardiac condition that presents in a variety of morphologies. Less commonly, when an individual patient is found to have multiple ventricular septal defects, the term “Swiss cheese ventricular septal defect” is applied. Although not routinely utilized in clinical practice, electrocardiogram (ECG)-gated computed tomographic angiography (CTA) has been shown to provide utility in detecting intracardiac shunts, demonstrating promise in preventing acute strokes secondary to a paradoxical embolus from occurring; this is especially important when atypical cardiac septa are suspected. This case seeks to illustrate how usage of ECG-gated CTA can assist in early detection and prevention of adverse outcomes resulting from an atypical presentation of a ventricular septal defect.

## Introduction

Ventricular septal defect (VSD) is one of the most commonly recognized congenital heart defects in patients and can often vary in location, size, and number.^1,2^ The interventricular septum is composed of a membranous septum and a muscular septum, and defects can be broadly categorized based on their morphological origin.^2^ A subset of patients with multiple small VSDs has been described, and the majority of such defects are found within the muscular portion of the interventricular septum.^3^ This phenomenon has been termed *Swiss cheese ventricular septal defect* (SCVSD).^3^

Uncommonly, VSDs may remain undetected in a patient for years or even decades due to lack of chronic signs or symptoms. In such patients, the initial presentation of an intracardiac shunt secondary to VSD can be an acute stroke, resulting from a paradoxical embolism from the right to the left side of the heart. Patients who suffer from such strokes should be evaluated thoroughly via echocardiography, the mainstay diagnostic tool for this situation. In cases where echocardiogram fails to localize the shunt, additional imaging with cardiac Magnetic Resonance Imaging (MRI) is usually warranted.

At present, use of electrocardiogram (ECG)-gating with computed tomographic angiography (CTA) is not recommended.^4^ Only a few investigators champion its routine use in the diagnosis of pulmonary embolism (PE).^5–7^ However, ECG-gated CTA is known to have utility in diagnosing intracardiac shunts, helping prevent one of the most catastrophic sequelae of PE, a paradoxical embolism secondary to an intracardiac shunt.^8–10^ A PE causes an abrupt change in right-sided heart pressure, which can reverse the direction of an intracardiac shunt instantly. Additional thromboemboli can then reach the left circulation and cause end-organ infarcts, with stroke being the most severe manifestation.^10^

The aim of this case is to illustrate the potential use of ECG-gated CTA for the diagnosis of PE and a concomitant intracardiac shunt in the setting of acute stroke. A challenging diagnosis of SCVSD was made incidentally during interpretation of an ECG-gated CTA, performed as follow-up of a known pulmonary embolus. The ECG-gated CTA results complemented those of a transthoracic echocardiographic bubble study, eliminating the need for cardiac MRI.

## Clinical presentation

A 55-year-old female was transferred to a tertiary center Emergency Department from a community hospital. Her chief complaints were: (1) shortness of breath and (2) right lower extremity (RLE) swelling for three days. During her visit to the community hospital, the patient was diagnosed with a deep vein thrombosis (DVT) and a saddle pulmonary embolus (PE) with right heart strain, and diagnoses were confirmed with venous duplex and CTA. Early during her hospitalization, her neurological status suddenly declined. The patient was noted to have a history of a cerebral vascular accident one month prior to her current illness, during which she presented with headache, expressive aphasia, and RLE weakness. She was not known to have any childhood history of congenital heart defects; additional medical history was unremarkable.

## Investigations

Computed tomography and angiography of the head and neck revealed a new, moderate-sized hemorrhagic infarct within the left cerebral hemisphere. Transthoracic echocardiogram (TTE) was then preformed, demonstrating a severely dilated right ventricle and right ventricular (RV) free wall dyskinesia, signs highly suggestive of acute PE. Evidence of RV pressure overload was confirmed with visualization of a flattened interventricular septum and elevated RV systolic pressure. Although no additional heart abnormalities could be identified on echocardiography, the patient’s recent history of stroke with concomitant PE findings continued to raise suspicion for an interventricular shunt. Consequently, the physician performing the examination decided to also perform a bubble study, which was positive for right-to-left shunting without Valsalva.

Because no intracardiac lesions or defects could be detected by the TTE, follow-up imaging was indicated. The patient was evaluated both by Neurology and Cardiology. Workup included a hypercoagulable panel, which was negative.

## Treatment and outcome

On the second day of her hospitalization, a heparin drip was started despite the patient’s stroke history, because of the life-threatening severity of the pulmonary embolus. Fortunately, the patient recovered from both her pulmonary embolus and stroke. A follow-up CT scan of the brain performed several days later showed that the intracerebral hemorrhage was stable. The patient was bridged to oral anticoagulant therapy and discharged eight days later. She was scheduled for neurosurgery and cardiology follow-up appointments as well as repeat imaging.

## Follow-up

A follow-up CTA PE scan was completed two months after discharge. ECG-gating was added to improve visualization of cardiac structures, as the initial echocardiogram was unable to definitively diagnose the cardiac pathology leading to a shunt. The scan was performed with a second-generation dual source CT scanner (Somatom Force, Siemens, Erlangen, Germany) at 120 kVp with a 300 mAs quality reference and both linear and ECG synchronized tube current modulation. 80 ml of Omnipaque 350 was injected at 5 mL/s followed by a 60-ml saline flush. Bolus-tracking was performed with a scan delay of 8 s utilizing a region of interest placed on the ascending aorta (rather than the pulmonary artery) with a threshold set to 100 Hounsfield units. (This protocol yielded adequate opacification of the left heart in addition to peak opacification of the pulmonary arteries). Images were reconstructed with 0.75-mm slice thickness, 0.5 mm interval, and a 220 mm displayed field of view, using the vendor’s proprietary soft cardiac kernel and iterative reconstruction algorithm.

**Figure 1.**
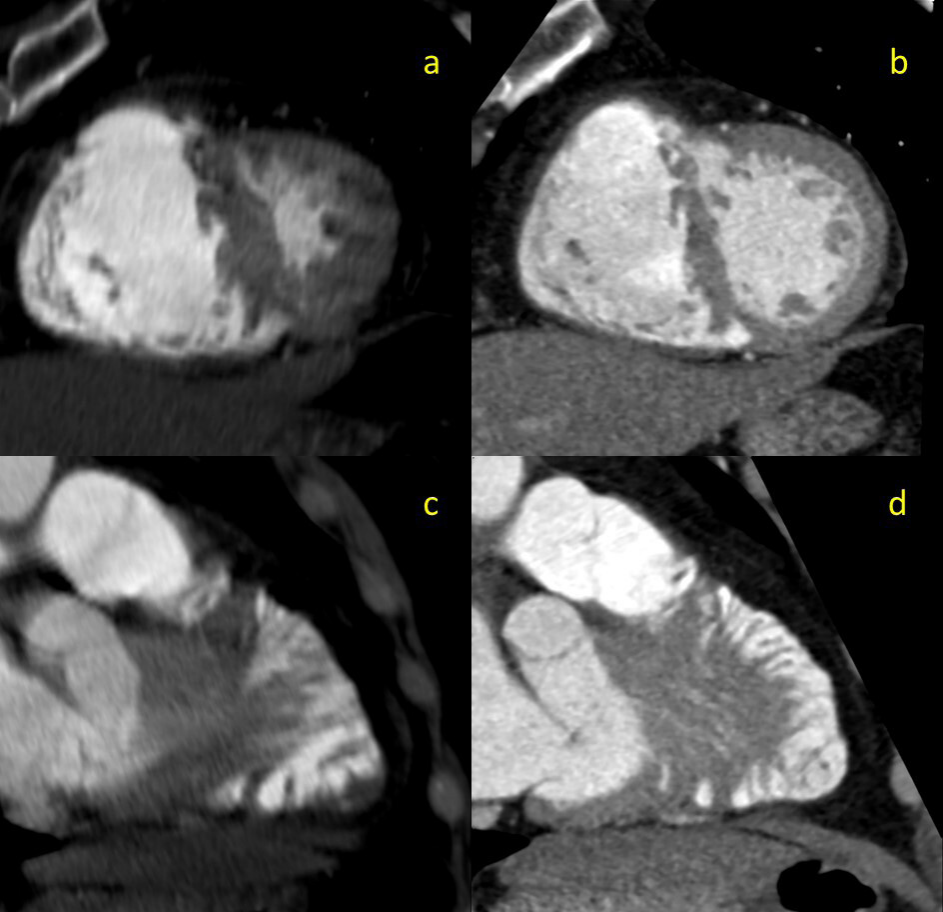
2-mm thick multiplanar reformation (MPR) images of the interventricular septum in cross-sectional (**a, b**) and lengthwise (**c,d**) orientations. Images on the left (**a,c**) are derived from the initial non-gated CTA. Images on the right (**b,d**) are derived from the follow-up gated-CTA exam

**Figure 2.**
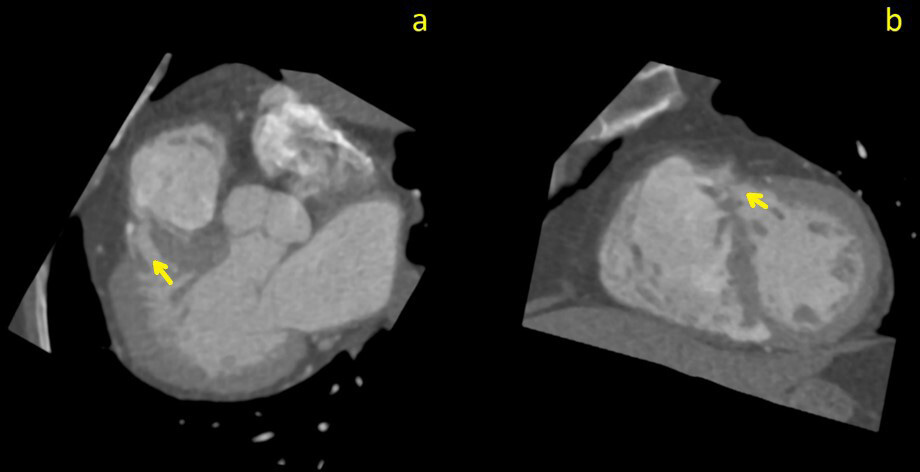
Curved planar reformation (CPR) images through the largest septal defect (arrows); images (**a**) pseudo-three chamber view; (**b**) pseudo-short axis view

This study showed that the PE resolved but also revealed a highly trabeculated right ventricle with associated “Swiss cheese” appearance of the septum (Figures 1b,d and 2), allowing for the diagnosis of SCVSD to be made. This finding was not discernable on the initial CTA, even retrospectively (Figure 1a,c). The anteroseptal and inferoseptal basal segments of the left ventricular myocardium exhibited multiple (*i.e.,* more than 3) small and large channels through the septum giving the septum a swiss cheese-like or spongy appearance.

## Discussion

Many factors played a role in the management decisions and care of this patient. Outside records showed evidence of lower extremity DVT as well as saddle PE with associated right heart strain. In the setting of recent stroke and imaging displaying an evolved focus of hemorrhage, the question of whether anticoagulation therapy should be used to treat the patient’s PE was key. Due to the possibility of potentiating the patient’s intracranial hemorrhage, anticoagulation was initially not recommended.

The stroke team initially recommended interventional thrombectomy and inferior vena cava (IVC) filter placement. However, the decision to commence with anticoagulation therapy was made by the Cardiology service who concluded that the risk of surgery was too great given the patient’s hemodynamic instability. The patient did well under conservative management, recovering from the PE without any further neurological decline.

2D echocardiography is the mainstay diagnostic modality for VSDs but does not always exclude the diagnosis. This is especially true if the patient has a muscular VSD due to heavy trabeculation of the muscular septum.^11^ Current guidelines recommend that patients with demonstrated cardiac shunting receive cardiac MRI after an inconclusive echocardiogram, as it has a higher resolution and offers functional information about shunt severity. However, concomitant findings of PE in this patient’s case made ECG-gated CTA a relevant and important diagnostic tool. Per institutional protocol, a CTA was indicated to monitor for resolution of the PE. Compared with non-gated techniques, ECG-gating has a comparatively higher radiation dose. However, in recent years, several guidelines and strategies have been adopted for dose reduction of ECG-gated imaging.^12^

The Congenital Heart Surgery Nomenclature database classifies VSDs into four types: subarterial (Type 1), peri-membranous (Type 2), inlet (Type 3), and muscular (Type 4). To make the diagnosis of SCVSD, a subcategory of Type 4 VSD, four or more muscular VSD’s must be identified on imaging.^13^ The diagnosis of SCVSD could not be made on the original CTA, even retrospectively. Cardiac motion creates artifacts on this modality, making assessment of intracardiac shunts impractical. In contrast, ECG-gated CTA, when performed correctly, is completely free of motion artefact. Given our demonstration of multiple VSDs on the follow-up scan, the use of ECG-gating confirmed the diagnosis and obviated the need for MRI. Although in this case, percutaneous closure of the multiple defects was never a consideration, in other scenarios involving intracardiac shunting (*e.g.,* a patent foramen ovale), early diagnosis could hasten a percutaneous closure. At those institutions where CT scanners with high temporal resolution and fast table speed have been installed, the use of ECG-gated CTA should be considered when patients present with signs of both PE and recent stroke if workup for other causes of stroke is non-diagnostic.

## Conclusion

In the setting of a patient presenting with DVT and PE with concomitant stroke, physicians must be wary of intracardiac shunts, including VSDs. Shunts are common and in the context of PE can lead to cryptogenic stroke due to paradoxical embolism. This case demonstrates the potential for ECG-gated CTA to assess for PE as well as both typical and atypical presentations of VSD. Thus, ECG-gated CTA, rather than CTA, may sometimes be the better test to assess for the pathology of pulmonary thromboembolic phenomena.

## Learning points

1. Swiss-cheese VSD is defined as four or more VSDs by the Congenital Heart Surgery Nomenclature database.
2. 2D echocardiogram may not exclude SCVSD’s as a diagnosis, due to heavy trabeculation of the muscular interventricular septum.
3. ECG-gated CTA offers a clear diagnostic advantage when evaluating for PE and atypical VSDs in the setting of a recent stroke.
